# Prostatic paracoccidioidomycosis with a fatal outcome: a case report

**DOI:** 10.1186/1752-1947-7-126

**Published:** 2013-05-13

**Authors:** Pedro Francisco Ferraz de Arruda, Márcio Gatti, José Germano Ferraz de Arruda, Daniel Cernach Ayres, Eduardo Maciel Narvaes, Laísa Ferraz de Arruda, Moacir Fernandes de Godoy

**Affiliations:** 1 Urology Department, São José do Rio Preto Medical School, Av. Brigadeiro Faria Lima 5416, 15090-000, São José do Rio Preto, SP, Brazil; 2 Medical Department, União das Faculdades dos Grandes Lagos, Unilago, Rua Dr. Eduardo Nielsem, 960 Jd. N. Aeroporto, 15030-070, São José do Rio Preto, SP, Brazil; 3 Cardiology and Cardiovascular Surgery Department, São José do Rio Preto Medical School, Av. Brigadeiro Faria Lima 5416, 15090-000, São José do Rio Preto, SP, Brazil

**Keywords:** Prostate, *Paracoccidioides brasiliensis*, paracoccidioidomycosis, prostatitis

## Abstract

**Introduction:**

Paracoccidioidomycosis is a systemic mycosis in Latin America that can affect various organs. Few case reports of paracoccidioidomycosis affecting the prostate are found in the literature.

**Case presentation:**

We present the case of a 79-year-old Caucasian man with a six-month history of irritative symptoms of the prostate (urgency, frequency and nocturia) and difficulty initiating urination that progressed to urinary retention and the use of a urinary catheter. The anatomopathological analysis of the transurethral resection of the prostate revealed chronic granulomatous prostatitis of fungal etiology (paracoccidioidomycosis) with extensive necrosis. The patient began treatment with itraconazole at a dose of 100mg/day for six months. Radiography of the thorax revealed bilaterally diffuse nodular reticular interstitial lesions. The patient progressed to respiratory failure and was sent to the intensive care unit, but suffered a cardiopulmonary arrest and was pronounced dead.

**Conclusions:**

Due to the high incidence of paracoccidioidomycosis in countries like Brazil, urologists should suspect blastomycosis in all patients with symptoms of lower urinary obstruction with chronic abacterial prostatitis. Considering that paracoccidioidomycosis has the potential to affect various organs, following diagnosis, the treatment must be initiated as soon as possible.

## Introduction

Paracoccidioidomycosis (South American blastomycosis) is a systemic mycosis in Latin America. Endemic among rural populations, paracoccidioidomycosis (PCM) mainly affects male individuals between 30 and 60 years of age, with rare occurrences in individuals under 14 years of age.

PCM is caused by a dimorphic fungus denominated *Paracoccidioides brasiliensis* and transmitted by means of the inhalation of spores through the respiratory system. The fact that dissemination of the parasite begins in the lungs and spreads through the lymphatic or hematogenic pathway leads to different presentations affecting any organ or system [1]. When located in the genital tract, the epididymis and testicles are the most frequently affected organs, followed in decreasing order by the prostate and penis [2]. Fungal prostatitis is rare. The most common agents of fungal prostatitis are *Candida albicans, Aspergillus sp., Cryptococcus neoformans* and *Blastomyces dermatitidis*, affecting mainly immunodepressed patients [1].

In the few studies involving the autopsy of patients with systemic dissemination of PCM, the rate of prostate involvement ranges from 2.7 to 9% [3,4]. Prostatitis by *P. brasiliensis* should be considered in the differential diagnosis for patients from endemic areas and persistent evidence of sterile pyuria, prostate nodules and lower urinary tract symptoms (LUTS) [5].

Few cases of PCM have been described with associated genital lesions [5,6] and significant irritative symptoms [1]. A review of the literature reveals few case reports of PCM affecting the prostate. The aim of the present paper is to describe just such a case.

## Case presentation

We present the case of a 79-year-old Caucasian male field worker from the state of São Paulo (Brazil) with a six-month history of irritative symptoms of the prostate (urgency, frequency and nocturia) and difficulty initiating urination that progressed to urinary retention and the use of a urinary catheter. The patient had a history of Chagas disease with cardiac impairment, coronary disease (a stent in his anterior interventricular artery) and dyslipidemia and sought the urology clinic for the evaluation of obstructive symptoms of the lower urinary tract.

A digital rectal examination revealed a prostate with an estimated size of 40cm^3^, slightly hardened at the apex, with no nodules and a prostate-specific antigen result of 1.2ng/ml. A physical examination revealed no alterations on the penis, testicles or epididymis. An unsuccessful attempt was made to remove the urinary catheter after initiating treatment with an alpha blocker (4mg). Thus, a transurethral resection of the prostate (TURP) was performed.

During his hospitalization for the TURP, the patient had the following hemogram: hemoglobin (Hb) 12.2g/mL; ematocrit (Ht) 36%; leukocytes 9300/mL; urinalysis with leukocytes 33,000 cells/mL; erythrocytes >500,000 cells/mL; negative uroculture; sodium 133mEq/L; potassium 4.3mEq/L; glucose 128mg/dL; and creatinine 1.4mg/dL. The patient remained in the intensive care unit in the immediate postoperative period at the recommendation of the cardiology team and was discharged from the infirmary asymptomatic, with no urinary catheter and a satisfactory urinary pattern.

**Figure 1:**
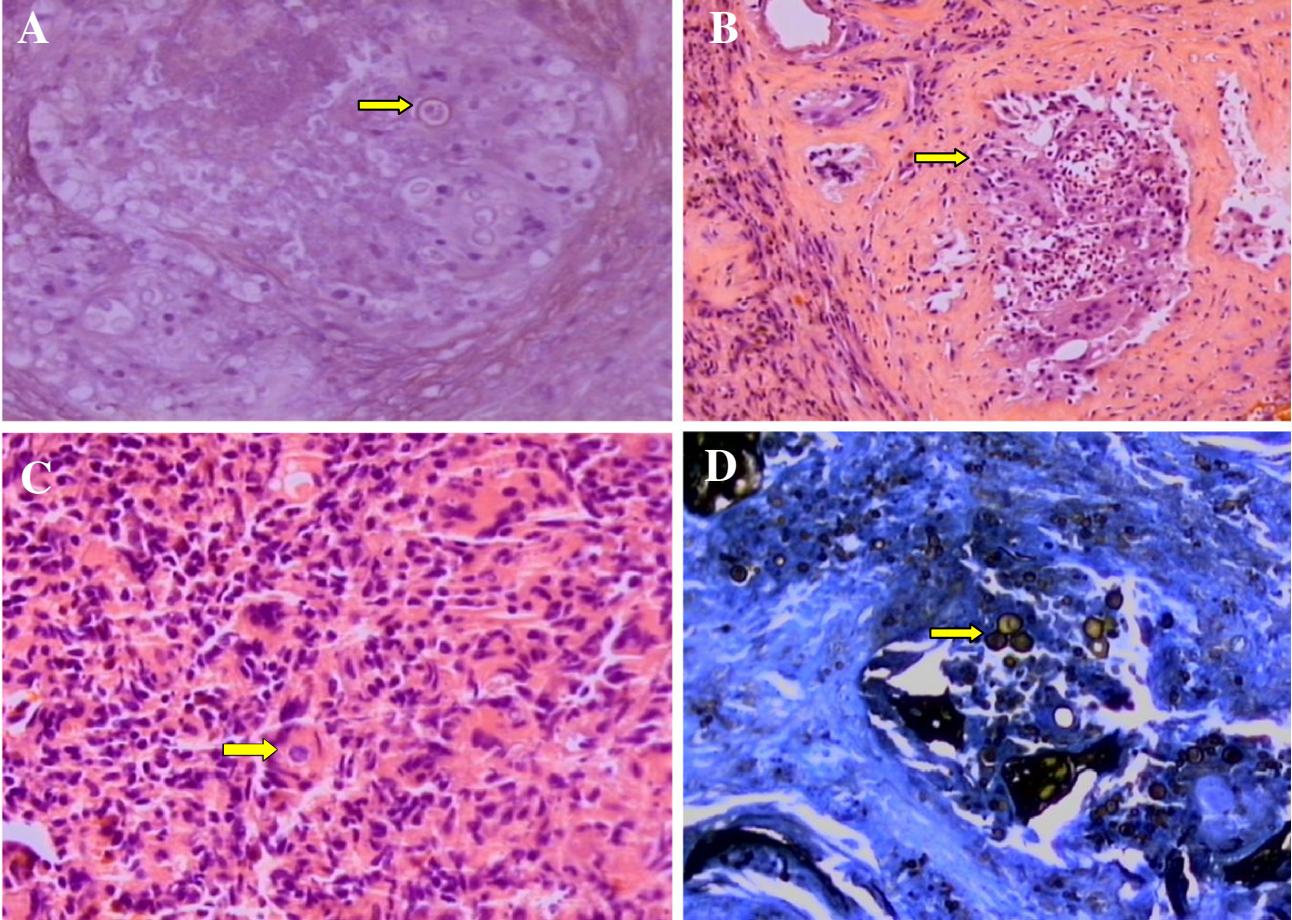
**Photomicrographs of prostate tissue showing.** (**A**) the chronic granulomatous inflammatory process with yeast fungi (arrow) (hematoxylin and eosin stain, 400×); (**B**) the chronic granulomatous inflammatory process with prostate acini in the upper left region (arrow) (hematoxylin and eosin stain, 100×); (**C**) the chronic granulomatous inflammatory process with yeast fungi (arrow) (hematoxylin and eosin stain, 400×); and (**D**) fungal cells with buds compatible with paracoccidioidomycosis (arrow) (Grocott’s methenamine silver stain, 400×).

Upon returning to the clinic, the anatomopathological analysis of the TURP revealed chronic granulomatous prostatitis of fungal etiology (PCM) with extensive necrosis (Figure 1A, B, C, D).

The patient was sent to the team specializing in infectious parasitic diseases and began treatment with itraconazole at a dose of 100mg/day for six months. On the occasion, a radiological evaluation was performed and revealed the involvement of both pulmonary lobes, with preservation of the apices.

The patient remained asymptomatic for three months, but was admitted to the emergency ward of the same hospital with intense weakness, dyspnea and vomiting. At the time, the patient exhibited leukocytosis (21,300 cells/mL), with 63% segmented neutrophils, a creatinine level of 6.3mg/dL and a C-reactive protein level of 26.7mg/dL.

**Figure 2:**
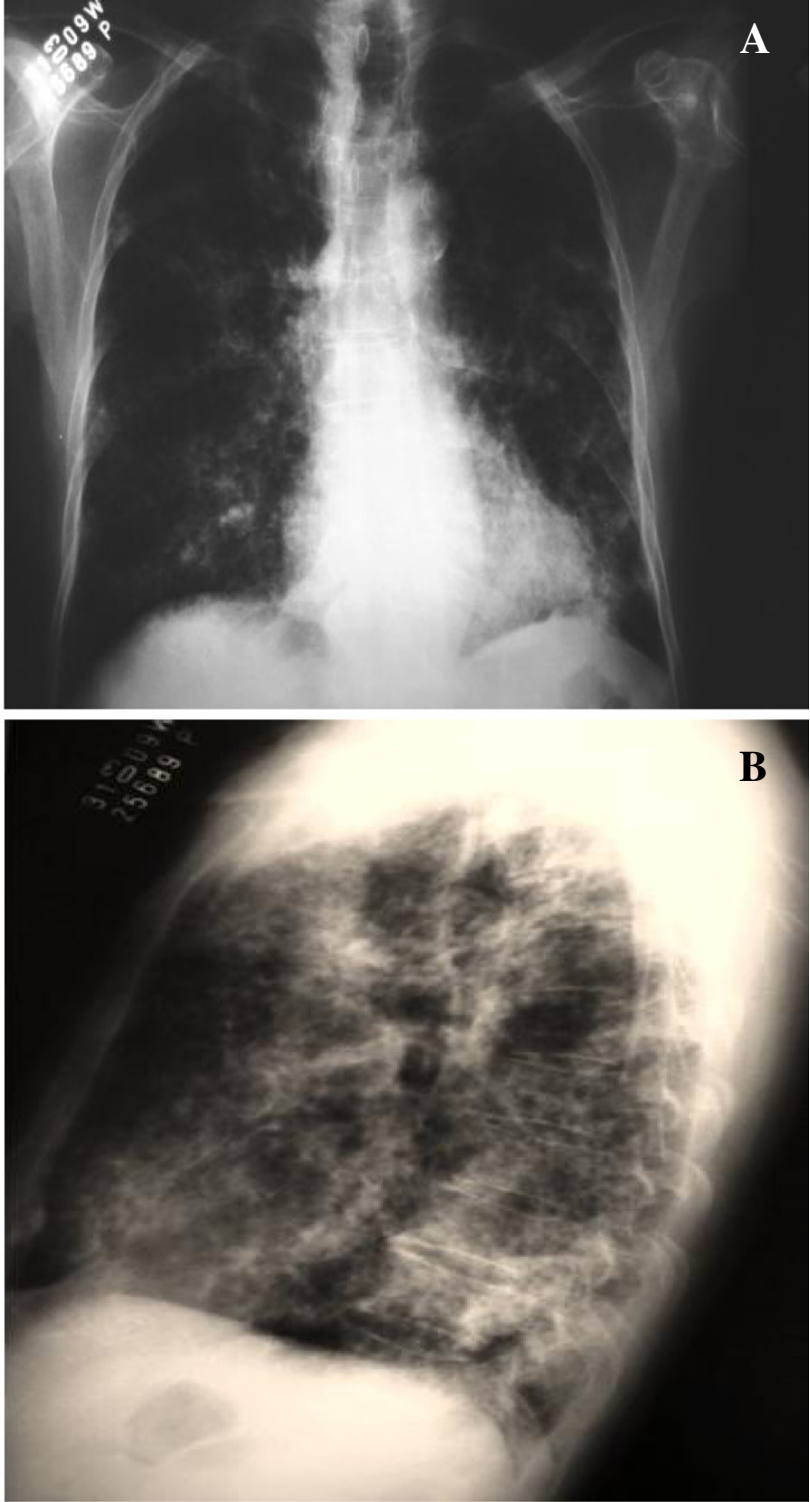
**Radiography of the thorax.** Anteroposterior view (**A**) and lateral view (**B**) showing bilaterally diffuse nodular reticular interstitial lesions.

Radiography of the thorax revealed bilaterally diffuse nodular reticular interstitial lesions (Figure 2A, B). Within a few hours, the patient progressed to respiratory failure and was sent to the intensive care unit, but suffered cardiopulmonary arrest and was pronounced dead just four hours after being admitted to the emergency ward.

## Discussion

PCM is a chronic granulomatous disease that typically affects the lungs, skin, bone, mucous membranes and lymph nodes [7]. Transmission occurs through the inhalation of spores. The most affected population is young adult farmers [8].

Due to its similarity to other influenza-like diseases during the acute phase, the diagnosis of PCM is generally not made. The chronic form of lung disease can progress to fibrosis and has typical radiological findings, as occurred in the patient described here. Due to the long latency period, late-onset manifestations of the disease may occur in the same period as the onset of prostate diseases [8].

Genitourinary impairment and consequences only affect a small proportion of patients with the disseminated form of the disease. Studies report contradictory figures ranging from 2.7 to 9% [3,4] and 1.59% of patients with the disseminated form of PCM and prostate involvement [9]. The first such case was described by Brito and Caprini in 1959 [10]. The entire genitourinary tract may be affected, including the epididymis, testicles, prostate, ureters, penis, urethra and kidneys.

As systemic mycosis is more common in Brazil, the differential diagnosis of sterile chronic prostatitis and prostate cancer in patients with lower urinary tract symptoms should be considered.

A positive culture of samples of bodily fluids and tissues confirms infection by *P. brasiliensis*. Immunodiffusion and complement fixation are the most frequently used serological tests, but are limited due to low sensitivity and specificity [11]. A chest X-ray should be performed on every patient with a diagnostic suspicion of such an infection [12].

The symptoms in patients with genitourinary system involvement include dysuria (painful urination), hesitance, nocturia, urinary retention, reduction in the urinary jet, suparpubic and perineal pain, hematuria and hemospermia [13]. There is a report of conjugal transmission, in which a man was diagnosed with PCM in the prostate and epididymis and his partner was diagnosed with infection by blastomycosis in the endometrium and uterine tube [14].

PCM is a systemic disease with the potential to affect various organs. Following diagnosis, the patient must be sent for evaluation to a specialist in infectious parasitic diseases. With regard to treatment, oral antimicrobial therapy is currently recommended, with the suggestion of itraconazole as the first line of defense, whereas the drug of choice is amphotericin B in patients with severe and/or immunodepressed symptoms [15]. There is no recommendation for fungal prophylaxis before the TURP. However, in patients from endemic areas with urinary retention and using a urinary catheter, the prophylactic treatment can be initiated before the TURP.

## Conclusions

Due to the high incidence of PCM in Brazil, urologists should suspect blastomycosis in all patients with symptoms of lower urinary obstruction with chronic prostatitis. The diagnosis may only be confirmed through an active search.

## Consent

Written informed consent was obtained from the patient’s next-of-kin for publication of this case report and any accompanying images. A copy of the written consent is available for review by the Editor-in-Chief of this journal.

## Competing interests

The authors declare that they have no competing interests.

## Authors’ contributions

PFFA contributed to the study conception and design, the acquisition, analysis and interpretation of the data, the drafting of the final manuscript, and the final approval of the version to be published. MG contributed to the acquisition and analysis of the data. JGFA contributed to the final approval of the version to be published. DCA contributed to the analysis and interpretation of the data. EMN contributed to the analysis and interpretation of the data. LFA contributed to the analysis and interpretation of the data and the drafting of the final manuscript. MFG contributed to the study conception and design, the analysis and interpretation of the data, the drafting of the final manuscript, and the final approval of the version to be published. All authors read and approved the final manuscript.
